# Potential of a deep eutectic solvent in silver nanoparticle fabrication for antibiotic residue detection

**DOI:** 10.3762/bjnano.15.38

**Published:** 2024-04-16

**Authors:** Le Hong Tho, Bui Xuan Khuyen, Ngoc Xuan Dat Mai, Nhu Hoa Thi Tran

**Affiliations:** 1 Faculty of Materials Science and Technology, University of Science, Ho Chi Minh City, Vietnam; 2 Vietnam National University, Ho Chi Minh City, Vietnam; 3 Center for Innovative Materials and Architectures (INOMAR), Ho Chi Minh City, Viet Nam; 4 Institute of Materials Science, Vietnam Academy of Science and Technology, Hanoi, Vietnam

**Keywords:** Ag NPs, antibiotic residue, deep eutectic solvents, potential, SERS

## Abstract

Deep eutectic solvents (DESs) have recently emerged as an alternative solvent for nanoparticle synthesis. There have been numerous advancements in the fabrication of silver nanoparticles (Ag NPs), but the potential of DESs in Ag NP synthesis was neither considered nor studied carefully. In this study, we present a novel strategy to fabricate Ag NPs in a DES (Ag NPs-DES). The DES composed of ᴅ-glucose, urea, and glycerol does not contain any anions to precipitate with Ag^+^ cations. Our Ag NPs-DES sample is used in a surface-enhanced Raman scattering (SERS) sensor. The two analytes for SERS quantitation are nitrofurantoin (NFT) and sulfadiazine (SDZ) whose residues can be traced down to 10^−8^ M. The highest enhancement factors (EFs) are competitive at 6.29 × 10^7^ and 1.69 × 10^7^ for NFT and SDZ, respectively. Besides, the linearity coefficients are extremely close to 1 in the range of 10^−8^ to 10^−3^ M of concentration, and the SERS substrate shows remarkable uniformity along with great selectivity. This powerful SERS performance indicates that DESs have tremendous potential in the synthesis of nanomaterials for biosensor substrate construction.

## Introduction

Surface-enhanced Raman scattering (SERS) is a ubiquitous technology for detecting and tracing substances, applied in various kinds of sensors. The spectra of SERS-based biosensors are simple but powerful results, in which every single component of the analytes can be recognized via characteristic vibrations of identical groups [1]. In particular, SERS is an advantageous and practical choice for biosensors in clinical settings thanks to fast response [2], the ability of real-time measurements [3], extremely high sensitivity [4], remarkable selectivity [5], and tremendous versatility [4,6–7]. Many scholars have taken advantage of these properties in cancer diagnosis [8], detection of hazardous chemicals [9], tracing of microorganisms [7,10–11], and other analytical measurements regarding food, medical, and environmental issues [12–14]. Undeniably, SERS is the future for sensor design.

So far, most achievements regarding SERS rely on the development of plasmonic materials. Noble metals (e.g., Au, Ag, and Cu) are the most important group of plasmonic materials, which extensively respond to electromagnetic waves with proper wavelengths in terms of free electrons resonating to the incident waves [9,15]. This is the fundamental principle of surface plasmon resonance (SPR). Moreover, plasmons are easily controlled at the nanoscale through different sizes, shapes, and surface morphologies of nanoparticles [16]. At the contacts among adjacent nanoparticles, so-called “hot spots” form; here, electromagnetic fields are effectively enlarged, leading to localized surface plasmon resonance (LSPR) [1,17]. Crucial parts of SERS-based biosensors are commonly made of LSPR materials [17]. With the development of synthesis techniques, numerous nanostructures of noble metals have been extensively studied to improve the intrinsic parameters of sensors.

Silver nanoparticles (Ag NPs) exhibit great performance in sensing applications owing to the best LSPR properties among the noble metals [18]. One of the decisive factors regarding the SERS performance of Ag NP-based platforms is the agglomeration state of the nanoparticles [19], which directly affects the “hot spots”. There have been many studies in which agglomeration of Ag NPs was adjusted by different kinds of surfactants such as cetyltrimethylammonium bromide [20–21], polyvinylpyrrolidone [18], and sodium dodecyl sulfate [21–22]. However, these chemicals have many negative effects on the environment including microbial, plant, soil, and marine ecosystems as reported by Rebello and co-workers [23]. This restricts the applicability of Ag NPs in the biomedical field and leads to the requirement for more eco-friendly products.

Recently, deep eutectic solvents (DESs) have been introduced to the chemical synthesis of nanomaterials. DESs show superior properties including high thermal stability, high polarity, low vapor pressure, and low toxicity, which makes DESs promising candidates for the replacement of thousands of industrial solvents [24–25]. DESs are so versatile that they have been used for nanomaterials synthesis [26–27]. Regarding plasmonic materials, gold nanoparticles (Au NPs) were the first to be fabricated in DESs [28–29]. SERS platforms based on Au NPs-DES whose sensitivity and durability are competitive to the other materials were successfully constructed [29–30]. However, no attention has been paid to the potential of DESs in the fabrication of Ag NPs. The similarities between Ag NPs and Au NPs, with the higher LSPR and SERS performance of Ag NPs [18,31], led to the innovative idea of Ag NPs synthesis in DESs.

In this work, we present a novel strategy to fabricate Ag NPs and demonstrate our hypothesis about the application of DESs in stabilizing Ag NPs. The resulting Ag NPs-DES is used for SERS detection of toxic antibiotics such as nitrofurantoin (NFT) and sulfadiazine (SDZ). These substances have been widely used since the 1970s because of rapid and absolute results against microbes [32], but they are also responsible for hormonal disruptions, methemoglobinemia, allergy, damaged liver, nausea, and cancer [33–36]. Despite these side effects, they are illegally overused in the food industry and medicine, which threatens the human food chain and negatively affects public health [37]. By evaluating the SERS parameters of the Ag NPs-DES substrate regarding the detection of NFT and SDZ, we demonstrate application aspects of our product, showing the great potential of DESs in sensing and biomedical applications.

## Results and Discussion

### Formation of Ag NPs-DES

**Figure 1:**
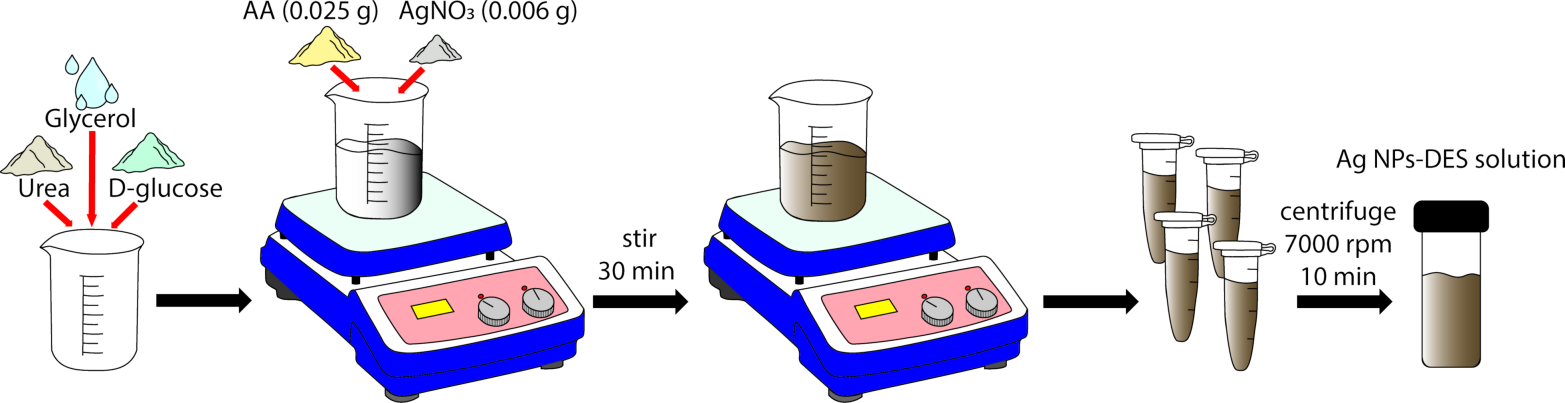
Schematic of the Ag NPs-DES synthesis.

**Figure 2:**
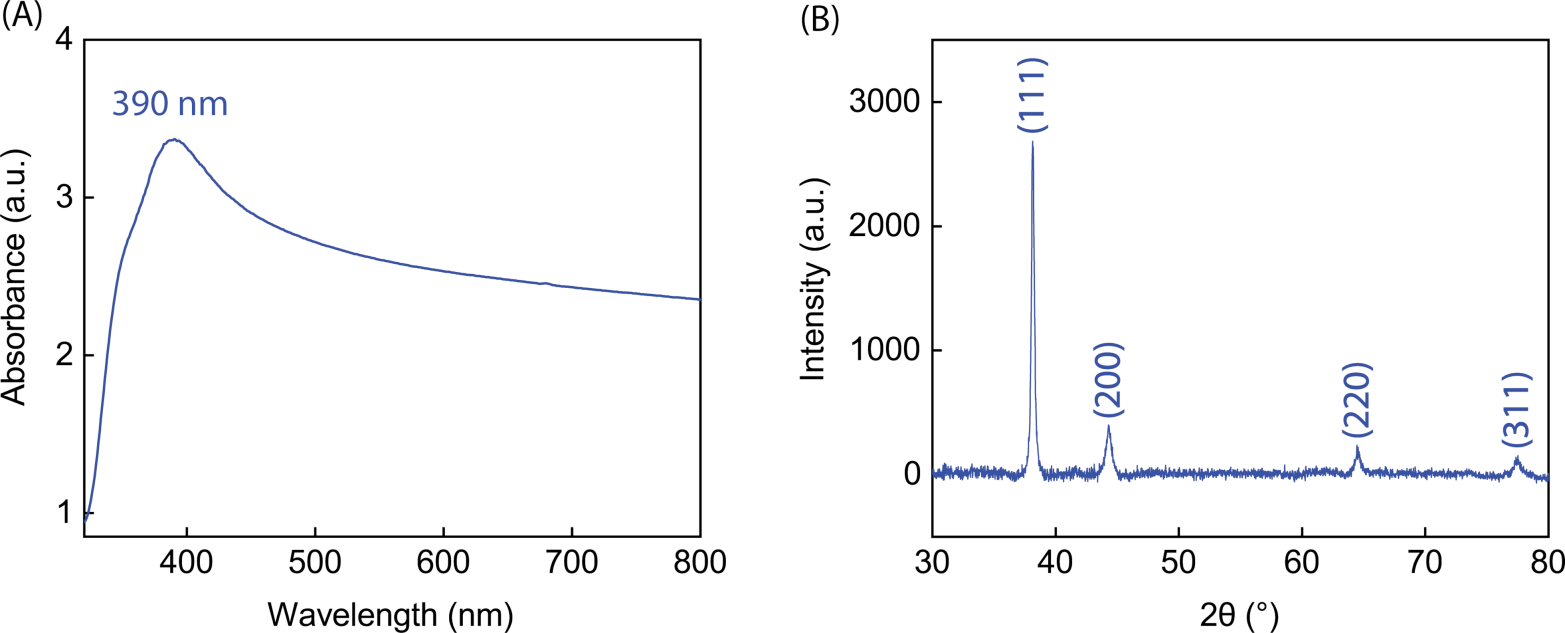
(A) UV–vis spectrum of the Ag NPs-DES solution. (B) XRD pattern of the Ag NPs-DES thin film.

We have developed new and simple strategy to fabricate Ag NPs-DES in which ascorbic acid was used as reducing agent. The synthesis protocol is summarily presented in Figure 1. Also, to characterize our material, UV–vis and XRD measurements were carried out. Figure 2A shows the broad adsorption band indicating the high number of excitons [38] on the surface of Ag NPs due to SPR. The SPR peak is located at 390 nm, which is suitable for SERS applications with 532 nm laser excitation. Besides, the shape of the UV–vis spectrum is in accordance with Mie scattering theory calculations, as reported in [39], proving the existence of Ag NPs in the solution. Moreover, the XRD pattern of the thin film (Figure 2B) shows four characteristic peaks at 38.2°, 44.3°, 64.4°, and 77.6°, corresponding to the (111), (200), (220), and (311) planes of face-centered cubic (fcc) Ag, respectively. The crystallite size is 30.61 nm, calculated from the most characteristic (111) peak of the Ag NPs [40]. From the presence of the fcc Ag lattice planes, we claim that Ag NPs-DES have been successfully synthesized [41].

**Figure 3:**
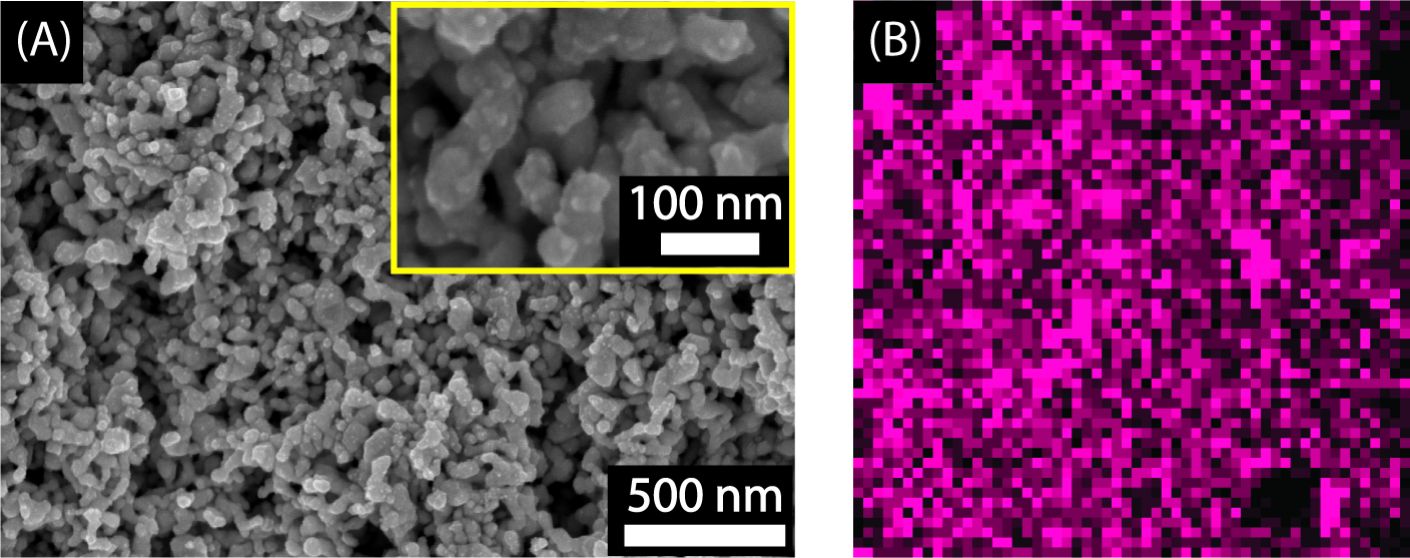
(A) SEM images of Ag NPs-DES. (B) XRF mapping of the Ag NPs-DES thin film with pink dots representing silver.

The development of clusters into nanoparticles following our strategy is supported by the DES. DESs have been reported to be potential shape-controlling agents, and highly branched nanostructures were the most common [30]. In our procedure, AgNO_3_ was added right after ʟ-ascorbic acid was dissolved in the DES at room temperature. The color of the mixture gradually turned from yellow-orange to dark brown, indicating the crystallization of Ag NPs. The synthesized Ag NPs-DES exhibits rod-like shapes of the NPs as well as a high aggregation state (Figure 3A). This is because of the high viscosity of the DES, which directly affected the stirring and yielded nonspherical NP shapes. The aggregated Ag NPs are supported by pure DES in which oxygen and hydrogen atoms of ᴅ-glucose, urea, and glycerol tend to form hydrogen bonds. The DES acts as a surfactant helping to stabilize Ag NPs. The rod-like appearance with 122.6 nm average length and small crystals on the surface of Ag NPs crucially contribute to strengthening the LSPR phenomenon thanks to the lightning rod effect [42]. As reported by other scholars, the rod-like morphology is better than a spherical one at increasing the extinction coefficient, about 10^9^ to 10^11^ M^−1^·cm^−1^ higher [43–44], which proves the applicability of Ag NPs-DES in SERS biosensors. Furthermore, X-ray fluorescence mapping was used to evaluate the presence of silver in the thin film (Figure 3B). The uniform distribution of silver shows the uniformity of Ag NPs-DES thin film on the glass substrate, which is crucial for the applicability of this material.

### NFT detection

**Figure 4:**
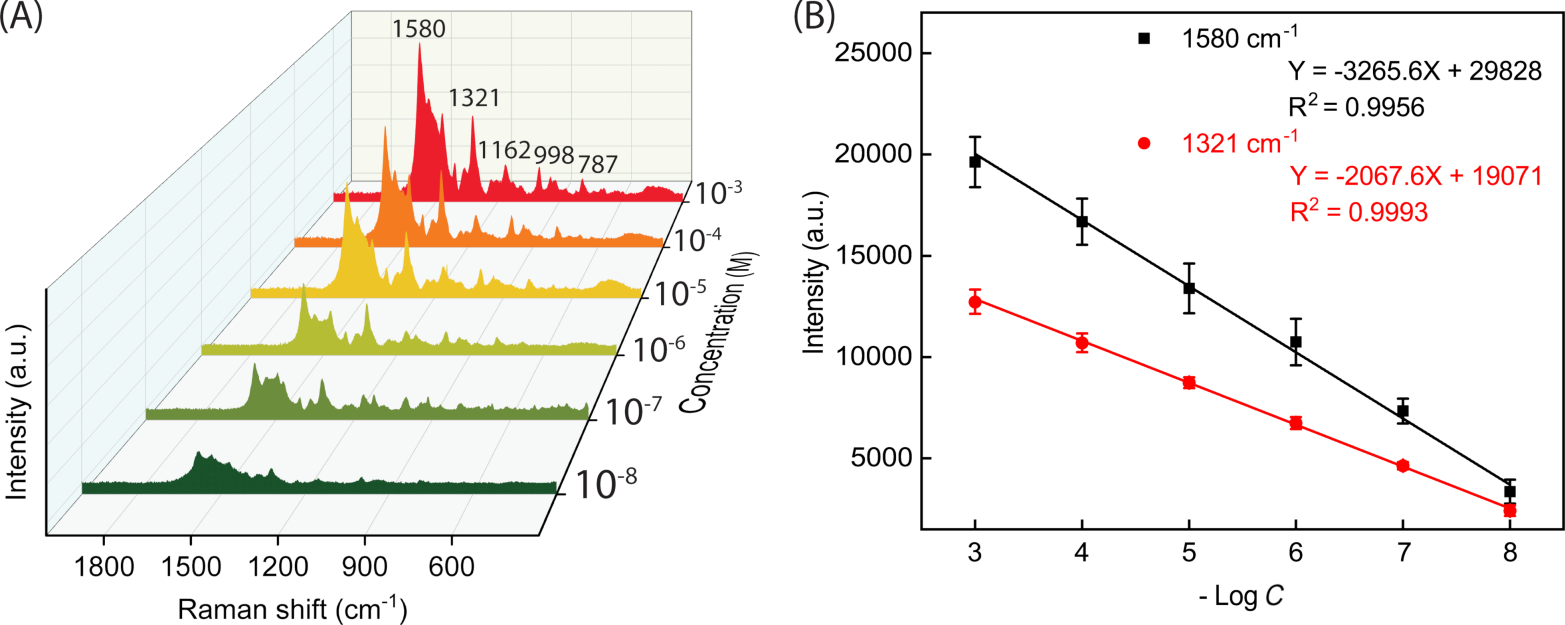
(A) SERS performance of the Ag NPs-DES substrate in detecting different concentrations of NFT. (B) Linear fit of −log *C* and peak intensities at 1580 and 1321 cm^−1^ (*C* stands for the concentration of NFT).

The most fundamental component of a SERS-based biosensor is its SERS substrate. It directly affects the SERS performance of the biosensor [6]. Herein, the Ag NPs-DES thin film with the superiorly uniform Ag NPs-DES coating can be used as a SERS substrate for the analysis of antibiotics. First, residue tracing of nitrofurantoin (NFT) has been conducted in the range from 10^−3^ M down to 10^−8^ M (Figure 4A). At the limit of detection (LOD) of 10^−8^ M, the SERS spectrum clearly shows emerging peaks, the highest enhancement factor (EF) of which reaches 6.29 × 10^7^, proving the NFT residue tracing capability of the Ag NPs-DES substrate. These peaks correspond to vibrations of characteristic groups of NFT as reported in Table 1 with the most intense ones at 1580 and 1321 cm^−1^. Consequently, these two peaks were used to construct the calibration curves as shown in Figure 4B. The *R*^2^ values for 1580 and 1321 cm^−1^ are 0.9956 and 0.9993, respectively, which is close to the ideal value of 1. This indicates that the Ag NPs-DES substrate is sensitive and can be used for quantitative analysis of NFT following the two linear fitting equations:


[1] $$I_{1580}=3265.6\log\left[\text{NFT}\right]+29828$$

[2] $$I_{1321}=2067.6\log\left[\text{NFT}\right]+19071$$

**Table 1:** Vibrational modes assigned to specific peaks in Raman spectra of selected antibiotics.

Chemicals	Frequency (cm^−1^)	Assignment^a^	Ref.

NFT	1580	ν N–N=C, ν –NO_2_	[45–46]
	1321	ω N–H	
	1162	ρ (furan ring)	
	998	ρ (hydantoin ring)	
	787	ν C–H	

SDZ	1567	ω N–H, ν (benzene ring)	[47]
	1356	ν C–N	
	1055	ν S=O	
	856	τ (pyrimidine ring)	
	610	ω (benzene ring)	

^a^ν: streching; ω: bending; τ: torsion; ρ: deformation.

**Figure 5:**
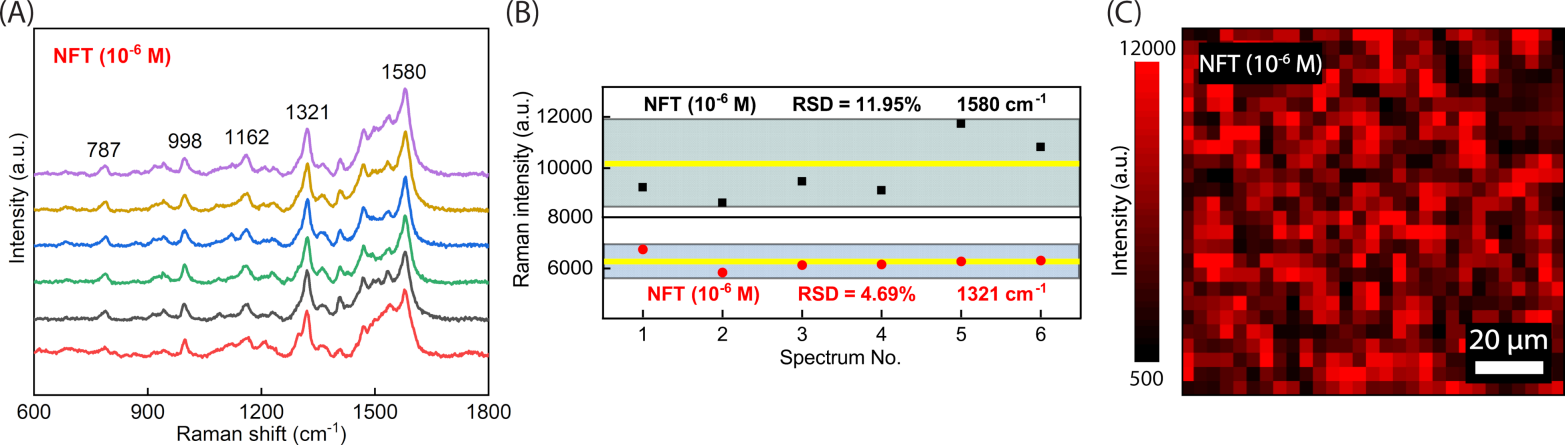
(A) SERS spectra at different points of the Ag NPs-DES substrate. (B) Variation of peak intensity at the two chosen wavenumbers. (C) SERS mapping of NFT (10^−6^ M) detected on the Ag NPs-DES substrate.

For further investigations on the SERS performance of the Ag NPs-DES thin film, NFT was again selected to test the stability. In practice, a stable SERS substrate is not only able to withstand the conditions of storage but also exhibits consistent Raman signals over the surface of the coating. Drops of 10^−6^ M NFT were placed on six different spots of the Ag NPs-DES substrate (Figure 5A). Then, the SERS spectra were analyzed by considering the variation of peak intensity of the two Raman peaks at 1580 and 1321 cm^−1^. In the diagram shown in Figure 5B, the yellow lines indicate the average intensity, while the areas covering all points represent the deviation. The peaks show a comparatively low relative standard deviation (RSD), namely 11.95% for 1580 cm^−1^ and 4.69% for 1321 cm^−1^. In addition, SERS mapping of 10^−6^ M NFT investigated on the substrate also helps to provide a larger scope on the uniformity of SERS signals. As shown in Figure 5C, there are plenty of high-intensity dots corresponding to signal enhancement by the Ag NPs-DES substrate, and the very even distribution indicates good uniformity, that is, satisfactory stability. These results can be explained by the LSPR of the rod-like Ag NPs synthesized in DES, which plays an important role in the high intensity [48] and the uniformity of the Ag NPs-DES coating proven in Figure 3B.

### SDZ detection and selectivity of Ag NPs-DES substrate

**Figure 6:**
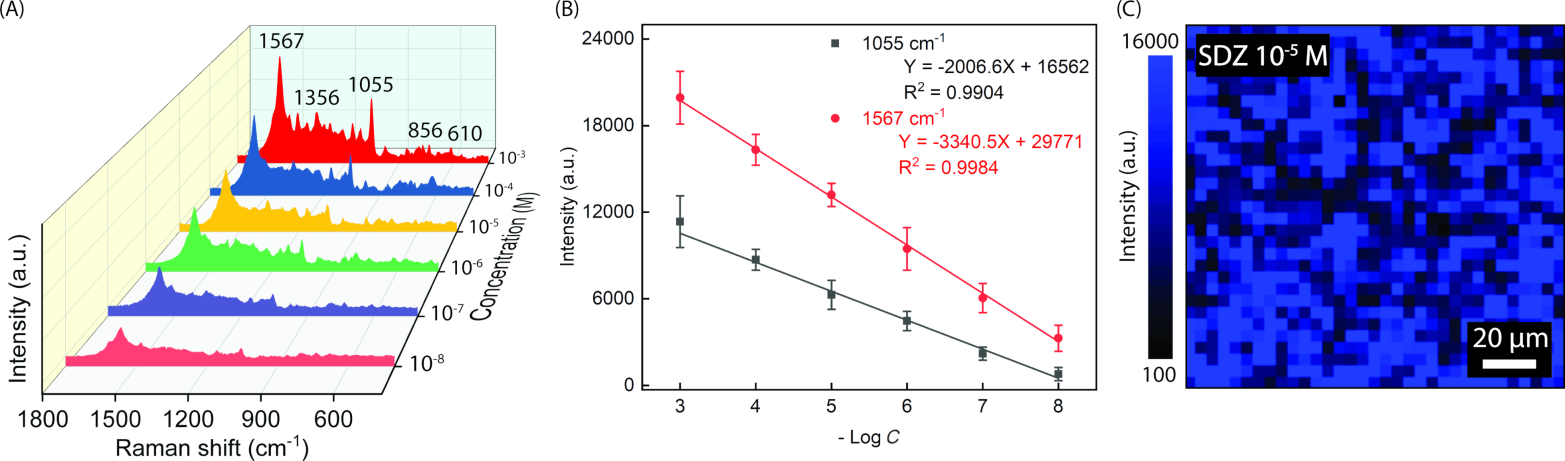
(A) SERS spectra of SDZ in the concentration range from 10^−3^ to 10^−8^ M. (B) Linear fit of SDZ concentrations and intensity of the peaks at 1055 and 1567 cm^−1^. (C) SERS mapping of 10^−5^ M SDZ on the Ag NPs-DES substrate.

Another antibiotic commonly used in infectious disease treatment is sulfadiazine (SDZ). Both NFT and SDZ are effective antimicrobial substances, but their overuse statuses were reported to be hazardous as mentioned above. Since their molecular structures are partially different [36,49], SERS analysis is meaningful for the selectivity test of the Ag NPs-DES substrate. In our study, different concentrations of SDZ were measured on the substrate to find out the LOD (Figure 6A). Collected peak intensity data were also used to construct the calibration curves shown in Figure 6B. The substrate shows a linear range from 10^−3^ to 10^−8^ M, in which the LOD value is 10^−8^ M, and the highest EF reaches 1.69 × 10^7^. The vibrational modes of the assigned peaks are listed in Table 1. There are two clearly enhanced peaks at 1567 and 1055 cm^−1^, whose correlation factors *R*^2^ are equal to 0.9984 and 0.9904, respectively. Along with the parameters of NFT SERS analysis obtained previously, these high *R*^2^ values help to provide undeniable proof of the remarkable quantity ability of our Ag NPs-DES substrate in antibiotic residue tracing. The linear regressions of the two chosen analytical peaks of SDZ are as follows:


[3] $$I_{1567}=3340.5\log\left[\text{SDZ}\right]+29771$$

[4] $$I_{1055}=2006.6\log\left[\text{SDZ}\right]+16562$$

Although the consistency in SERS signals recorded on the Ag NPs-DES substrate has been investigated with 10^−6^ M NFT, we need to evaluate the SERS mapping image of SDZ to ensure the stability of our substrate when analytes are changed. Hence, 10^−5^ M of SDZ was added dropwise and let dry naturally before the laser excitation. The SERS mapping shown in Figure 6C shows an even distribution of high intensity over the entire considered surface. This shows that the Ag NPs-DES coating has good consistency despite the different analytes. DES is supposed to play a crucial role in dispersing the Ag NP suspension via its hydrogen bonding networks [30], which increases the possibility of linkage formation between –NH_2_ groups of 3-aminopropyl)triethoxysilane (APTES) and Ag NPs. This eventually explains the evenness of the Ag NPs-DES thin film.

**Figure 7:**
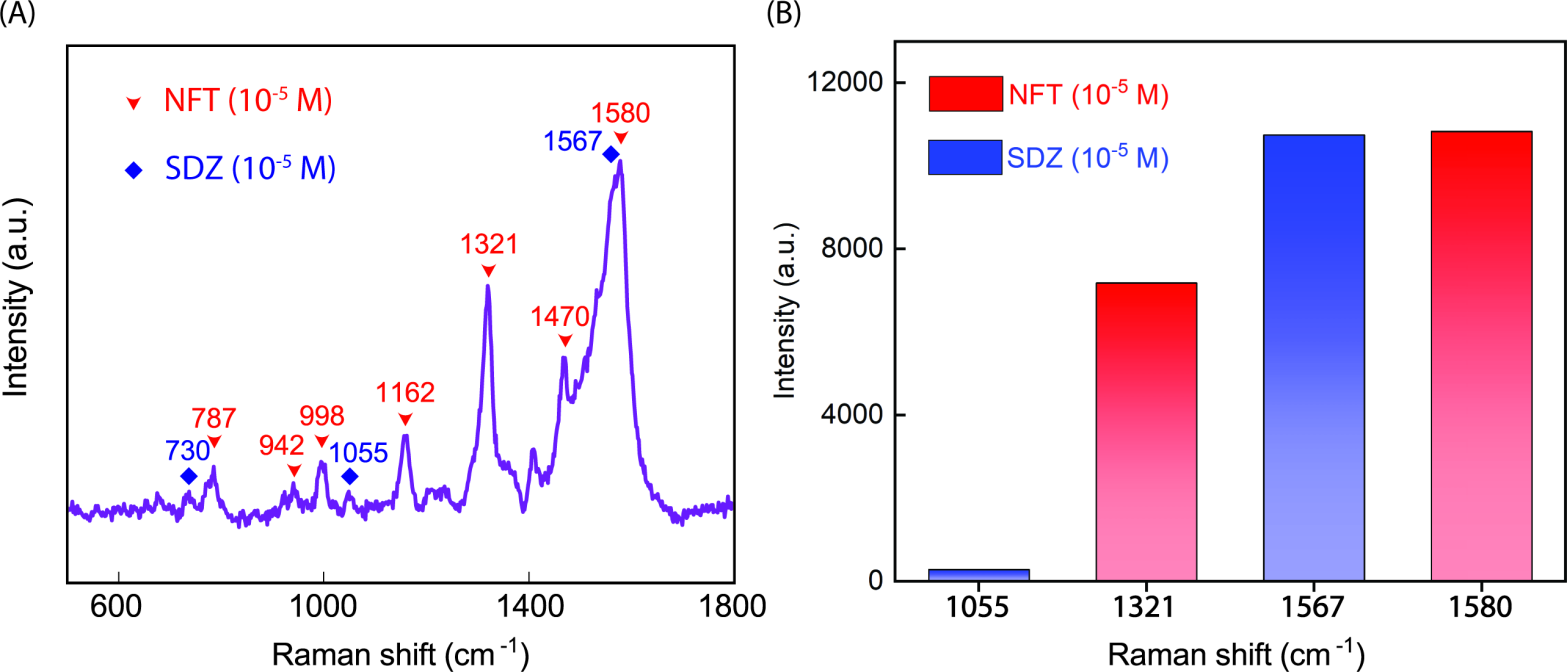
(A) SERS spectrum of NFT (10^−5^ M) and SDZ (10^−5^ M) solution. (B) Specific peak intensity identification corresponding to NFT and SDZ.

Another type of selectivity test was carried out with a solution containing 10^−5^ M NFT and 10^−5^ M SDZ. The SERS spectrum in Figure 7A verifies the difference in Raman shifts among the characteristic peaks of the two analytes. Here, the blue diamonds represent SDZ’s key peaks, whereas the red arrows stand for the ones of NFT. With the presence of all characteristic peaks, NFT is effortless to detect in the solution thanks to the high intensity. In contrast, the SERS spectrum of SDZ shows solely three peaks instead of the common five. Additionally, the two most intense peaks of NFT at 1580 cm^−1^ and SDZ at 1567 cm^−1^ overlap, which makes it difficult to ascertain the intensity for quantitative determination (Figure 7B). Therefore, we propose the peaks at 1321 cm^−1^ of NFT and 1055 cm^−1^ of SDZ as alternatives. Their intensity is comparable to the base of the SERS spectrum, and they are separated from each other as well as from the others. Based on the data experimentally collected and the correlation between −log *C* and peak intensity as given in Equation 2 and Equation 4, the concentrations of NFT and SDZ can be determined. This surely can be applied to other solutions of various substances, indicating that the Ag NPs-DES substrate has a good selectivity. Moreover, compared to other studies on NFT and SDZ detection (Table 2), the Ag NPs-DES material shows competitive LOD values and a linear range. Thus, Ag NPs-DES is a promising candidate in SERS applications along with the tremendous potential of DES in Ag NPs fabrication.

**Table 2:** An overview of reported studies on NFT and SDZ detection.

Analyte	Material	Year	LOD	Linear range	Ref.

NFT	Au NPs/GO	2021	5 ng/mL	5–500 ng/mL	[46]
	BT/CNF/GCE	2021	5 × 10^−9^ M	4.5 × 10^−4^ to 6 × 10^−8^ M	[50]
	N-CQD@Co_3_O_4_/MWCNT	2020	4.4 × 10^−8^ M	1.22 × 10^−3^ to 5 × 10^−8^ M	[51]
	NiO/BCN	2019	10^−8^ M	2.3 × 10^−4^ to 5 × 10^−8^ M	[52]
	Ag NPs-DES	2023	10^−8^ M	10^−3^ to 10^−8^ M	this work

SDZ	SrWO_4_	2021	9 × 10^−9^ M	2.35 × 10^−4^ to 5 × 10^−8^ M	[53]
	Au NPs	2021	1 µg/L	1–100 µg/L	[49]
	RGO/Ag - coated alloy fiber	2019	1.9 ng/cm^3^	0.01–100 µg/cm^3^	[54]
	MIP - QDs	2019	6.7 × 10^−7^ M	4 × 10^−6^ to 2 × 10^−5^ M	[55]
	Ag NPs-DES	2023	10^−8^ M	10^−3^ to 10^−8^ M	this work

## Conclusion

In this study, we have proposed a novel strategy for Ag NP synthesis in a DES composed of ᴅ-glucose, glycerol, and urea. The Ag NPs-DES sample was prepared successfully through chemical reduction, in which DES acts as solvent and shape-controlling agent. Using NFT and SDZ as probe molecules, the SERS performance of Ag NPs-DES was discussed. The LOD value is 10^−8^ M for the detection of NFT and SDZ. The EF values are relatively high, 6.29 × 10^7^ for NFT and 1.69 × 10^7^ for SDZ, and the linearity coefficients are very close to 1, proving the quantitative residue tracing capabilities of the synthesized Ag NPs-DES substrate. Besides the high sensitivity, the sensor exhibits also uniformity of the coating, consistency in SERS signals, and good selectivity. Overall, Ag NPs-DES is a promising candidate for SERS-based biosensor applications. This work hopefully provides useful information about the potential of DES in nanomaterials fabrication and a possible guidance for low-cost and effective SERS substrate construction in biosensors.

## Experimental

### Chemicals

ʟ-Ascorbic acid (AA, C_6_H_8_O_6_, 99%), silver nitrate (AgNO_3_, 99%), (3-aminopropyl)triethoxysilane (APTES, 99%), NFT (C_8_H_6_N_4_O_5_, 98%), and SDZ (C_10_H_10_N_4_O_2_S, 99%) were purchased from Sigma-Aldrich Co., MO, USA. Urea (CH_4_N_2_O, 99%) was obtained from ACS, Reag. Ph Eur, Merck Co., Germany, whereas glycerol (C_3_H_8_O_3_, 99%) was supplied by Daejung Ltd., Korea. ᴅ-glucose (C_6_H_12_O_6_, 99%) was purchased from Fisher Ltd., UK. The microscope glass slides (SiO_2_, Na_2_O, CaO, and MgO) were manufactured by ISOLAB Laborgeräte GmbH, Eschau, Germany.

### Fabrication of DES and Ag NPs-DES

The most ubiquitous DES studied vastly in recent years is reline, composed of urea and choline chloride in a 2:1 molar ratio [24]. However, this substance is not appropriate for Ag NPs synthesis because of the Cl^−^ anions in choline chloride, which may unintentionally cause AgCl precipitation. Here, we chose an alternative DES [56] without any anions precipitating with Ag^+^ cations. ᴅ-glucose, urea, and glycerol (molar ratio 1:1:2) were mixed and magnetically stirred at relatively high temperature until a homogenous liquid formed. Then, the mixture was cooled down gradually to room temperature while keeping the vigorous stirring. 0.025 g of AA was dissolved in 10.195 g of fabricated DES, and 0.006 g of AgNO_3_ was added later, which helped the reaction to occur. After 30 min of constant stirring, the obtained Ag NPs-DES were washed with DI water several times, and the pellets were re-dispersed in DI water for further use.

The Ag NPs-DES thin film was prepared following the procedure for self-assembly monolayer construction. A clean glass substrate was treated with oxygen plasma to form reactive –OH groups on the surface. The substrate was then soaked in a 3% ethanolic solution of APTES for 2 h, which helped to stabilize the –NH_2_ groups. A total of 2 mL of Ag NPs-DES solution was used to deposit Ag NPs on the glass substrate via Ag–NH_2_ linkage by fully immersing the treated glass for 2 h. At last, the product was dried naturally at room temperature, resulting in the successful fabrication of the Ag NPs-DES substrate.

### NFT and SDZ detection on the Ag NPs-DES substrate

Various concentrations from 10^−3^ to 10^−8^ M of NFT and SDZ were added dropwise on the Ag NPs-DES substrate (20 µL for each measurement). The analyte was then dried at room temperature, and the Raman spectra were recorded via laser excitation at 532 nm. Other investigations on the SERS performance of our sample were also carried out, including Raman mapping, signal consistency, uniformity, and selectivity.

### Instrument characterization and apparatus

The absorbance properties of the sample were recorded using a V-730 UV–vis–NIR spectrophotometer supplied by JASCO, Japan. The crystallinity of the Ag NPs-DES thin film was determined using a D8 Advance diffractometer, Bruker, UK, with a Ni-filtered Cu Kα X-ray source. To evaluate the nanostructure and surface morphology of the nanoparticles, as well as the elemental distribution of silver on the substrate, a S4800 field-emission scanning electron microscope purchased from Hitachi, Japan, and an M4 TORNADO^Plus^ Micro X-ray fluorescence spectrometer with a Rh tube at 30 W micro-focus light element from Bruker, UK, were used. Raman spectra were collected with a XploRA ONE spectroscope (HORIBA, Japan), with a laser wavelength of 532 nm, 1 mW power, and an accumulation number of 60.

## Data Availability

The data that supports the findings of this study is available from the corresponding author upon reasonable request.
